# Comparative genomics reveals hidden biosynthetic diversity in *Streptomyces spp*. and metal-dependent regulatory features associated with untapped specialized metabolites

**DOI:** 10.3389/fmicb.2026.1928796

**Published:** 2026-08-27

**Authors:** Nada S. Al-Theyab, Haila M. Alnassar, Mohanad A. Ibrahim, Kotb A. Attia

**Affiliations:** 1 Department of Pharmaceutical Chemistry, College of Pharmacy, King Saud University, Riyadh, Saudi Arabia; 2 Advanced Diagnostics & Therapeutics Institute, Health Sector, King Abdulaziz City for Science and Technology (KACST), Riyadh, Saudi Arabia; 3 Data Science Program, King Abdullah International Medical Research Center, Riyadh, Saudi Arabia; 4 Center of Excellence in Biotechnology Research, King Saud University, Riyadh, Saudi Arabia

**Keywords:** comparative genomics, cryptic biosynthetic gene clusters, genome mining, metal-responsive regulators/ metal-responsive repressor regulators, secondary metabolites, streptomyces spp

## Abstract

The genus *Streptomyces* is one of the richest sources of bioactive natural products; however, a substantial proportion of its biosynthetic gene clusters (BGCs) remain cryptic and their metabolic products are unresolved. Advances in genome mining and computational prediction now enable comprehensive exploration of this hidden biosynthetic repertoire. In this study, whole-genome sequencing and comparative genomic analyses were performed on three three newly isolated *Streptomyces* strains to evaluate their specialized metabolic potential. Genome assemblies were annotated and systematically analyzed using antiSMASH, DeepBGC, GECCO, and PRISM to identify, cross-validate, and functionally characterize BGCs while predicting their associated secondary metabolite scaffolds. Taxonomic analyses based on Average Nucleotide Identity (ANI), phylogenomics, and BLAST identified the isolates as *Streptomyces thinghirensis, Streptomyces novocaesareae*, and *Streptomyces griseorubens*. Applying the consensus framework across the three *Streptomyces* genomes yielded 43 cryptic BGCs, lacking close similarity to reference BGCs in the MIBiG database, of which 26 were classified as HIGH, 10 as MEDIUM, and 7 as LOW confidence. Notably, numerous BGCs exhibited low abundance to characterized reference clusters, indicating a high potential for previously undescribed biosynthetic pathways and novel metabolite scaffolds. Comparative analyses further revealed strain-specific biosynthetic architectures together with putative metal-responsive regulatory systems; *Fur, Zur*, and *Nur*, which were frequently associated with specialized metabolite biosynthetic loci. Collectively, these findings demonstrate the effectiveness of integrated genome-mining strategies for prioritizing cryptic biosynthetic gene clusters and highlight the remarkable biosynthetic potential of newly identified *Streptomyces* isolates as a source of novel natural products.

## 1 Introduction

*Streptomyces* remain major producers of clinically important natural products. However, genomic and metagenomic studies reveal numerous biosynthetic gene clusters (BGCs), whose associated metabolites are typically not detected under routine laboratory cultivation conditions highlighting a large reservoir of cryptic biosynthetic capacity with therapeutic potential (Liu et al., 2021). Focusing on enzymes that install distinctive functional groups can make genome mining more targeted, especially when prioritizing orphan BGCs that encode these motifs, which may yield new bioactive molecules (Ochi and Hosaka, 2013). Characterizing the corresponding biosynthetic pathways clarifies the underlying chemistry and supports the rational design of analogs with modified or novel scaffolds (Ochi and Hosaka, 2013).

Combining genome mining with detailed biosynthetic pathway therefore offers a powerful route to discover innovative natural products (Jeong et al., 2025). This framework distinguishes four situations in secondary metabolism based on what is known about BGC and its metabolite. When a BGC is identified but its metabolite has not been detected, the pathway is considered cryptic or silent; conversely, when a metabolite is known but the responsible BGC has not been assigned, the compound itself is cryptic because its biosynthesis is unresolved (Hoskisson and Seipke, 2020).

Cryptic BGCs that have been identified but not linked to known secondary metabolites, as they remain undetected under standard conditions (Cook and Stasulli, 2024). Once such clusters are located in the genome, bioengineering strategies can be used to activate their expression or alter their products, thereby enhancing or diversifying natural product output. Notably, many cryptic BGCs arise from non-ribosomal peptide synthetase (NRPS) and polyketide synthase (PKS) pathways, which encode complex enzyme systems responsible for amino acid–derived natural products (Cook and Stasulli, 2024).

Approaches based on synthetic biology, cell signaling, and stress induction are frequently employed to activate these clusters for novel molecule discovery (Rutledge and Challis, 2015). Recent advances in genome engineering, including CRISPR/Cas systems, PCR-targeting, transformation-associated recombination (TAR) cloning, and Cre/loxP-mediated recombination, have significantly improved the genetic engineering of *Streptomyces*, enabling efficient activation of cryptic BGCs and optimization of secondary metabolite biosynthesis (Krysenko, 2025). Nevertheless, a deeper mechanistic understanding of silent metabolism in *Streptomyces* is still required to fully exploit their biosynthetic potential and help close the drug discovery gap (Qi et al., 2021; Rigali et al., 2018). These findings highlight promoter architecture and network-level regulation as key contributors to biosynthetic crypticity and provide concrete targets for rational activation of silent BGCs in drug discovery (Qi et al., 2021).

Regulatory-guided genome mining has emerged as a promising strategy for cryptic BGC discovery. By exploiting transcription factor binding sites and regulatory networks, previously overlooked pathways can be identified and prioritized (Augustijn et al., 2024). For example, targeting the zinc regulon through the zinc-responsive regulator (*Zur*) enabled the discovery of a novel zinc-associated gene cluster, demonstrating the utility of regulatory information for BGC elicitation and natural product discovery (Augustijn et al., 2024; Spohn et al., 2016). Metal-responsive repressors are important regulators of metal homeostasis in *Streptomyces*. In *S. griseus*, nickel-responsive repression of *sodF* is controlled by *SrnR* and the nickel-binding protein *SrnQ* (Kim et al., 2003). Similarly, nickel-responsive regulation has been reported in *S. griseus*, where expression of the *sodF* gene is controlled through a specific cis-regulatory operator that mediates transcriptional repression in response to nickel availability (Kim et al., 2000). Yang et al. (2022) showed that *Zur* activates transcription in *S. coelicolor* through interactions with Zur-box elements and RNA polymerase, providing a framework for understanding Fur-family regulation.

A broad suite of computational tools now supports genome mining of secondary metabolite BGCs, ranging from sequence-search platforms to dedicated predictors such as Antibiotics and Secondary Metabolite Analysis Shell (antiSMASH) that classify many cluster types using Hidden Markov Models (HMMs) and curated rules (Blin et al., 2025). Recent machine-learning tools, including A deep learning genome-mining strategy for biosynthetic gene cluster prediction (DeepBGC), Gene Cluster prediction with Conditional Random Fields (GECCO), improve the detection of atypical and novel biosynthetic gene clusters, and PRediction Informatics for Secondary Metabolomes (PRISM) for expanded prediction of natural product chemical structures from microbial genomes (Hannigan et al., 2019; Carroll et al., 2021; Skinnider et al., 2017).

Applying these pipelines to *Streptomyces* has revealed that each genome encodes numerous, often cryptic BGCs. underscores both the genus's underestimated biosynthetic potential and the need for improved prediction and experimental validation (Blin et al., 2025; Lobo, 2008). Most forward genome-mining studies in *Streptomyces* relied on antiSMASH, which was repeatedly applied across multiple of several *Streptomyces spp.*, leading to the discovery of diverse metabolites such as peptides, polyketides, and antibiotics. BLASTP was commonly used for homology-based mining in similar strains while some studies combined antiSMASH with BLAST to improve cluster validation (Lee et al., 2020). Overall, antiSMASH remains the dominant platform for BGC bioactive metabolite discovery in *Streptomyces* (Blin et al., 2025).

The present study focuses on three newly isolated *Streptomyces* strains identified as *S. thinghirensis, S. novocaesareae*, and *S. griseorubens*. These taxonomically distinct isolates form the basis of a comparative genome mining analysis to investigate their cryptic biosynthetic gene cluster repertoires and metal-responsive regulatory systems associated with secondary metabolite biosynthesis.

Recent studies have highlighted the therapeutic potential of *S. thinghirensis S. novocaesareae* and *S. griseorubens*. Metabolites from *S. thinghirensis* strains have demonstrated antimicrobial, antioxidant, and anti-inflammatory activities (Osman et al., 2024). While *S. griseorubens* has been reported to produce compounds with antioxidant and cytotoxic properties (Rasmey et al., 2024). In contrast, *S. novocaesareae* remains comparatively underexplored, with limited information available regarding its specialized metabolism and biosynthetic capacity.

This genomic investigation of these three *Streptomyces spp*. aimed to compare the predictive capabilities of antiSMASH, DeepBGC, and GECCO for cryptic biosynthetic gene cluster discovery, characterize their repertoires, assess strain-specific biosynthetic diversity through PRISM-based genomic prediction of secondary metabolomes, and identify putative metal-responsive regulatory systems associated with secondary metabolism.

By integrating comparative genomics and genome mining approaches, we uncovered cryptic biosynthetic pathways linked to metal-responsive repressor regulators, highlighting an untapped reservoir of biosynthetic diversity for the discovery of novel bioactive compounds and previously uncharacterized analogs.

## 2 Methods

### 2.1 Bacterial strains and culture conditions

The *Streptomyces* strains examined in this study were collected from agricultural soils on two university campuses in Egypt: Assiut University in Upper Egypt and Kafr El-Sheik University in the Nile Delta. Both sites were post-harvest wheat fields, selected to represent actively cultivated agricultural soils typical of the region.

Following purification, the isolates were preserved at −80°C for long-term storage. Cultures revived using a sterile inoculating loop and streaked onto Mueller–Hinton agar (MHA; Oxoid, UK) in sterile Petri dishes (MHA) were selected due to its ability to support robust growth and reliable morphological characterization of *Streptomyces spp*., and its successful application in previous studies (Mazumdar et al., 2023). All cultures were incubated at 36°C—aerobic conditions for 7 days under standardized conditions. To ensure reproducibility and purity, each isolate sub-cultured in biological replicates. The purification procedure was repeated three times through single-colony isolation to eliminate potential contamination.

### 2.2 Genomic DNA extraction and quality assessment

Genomic deoxyribonucleic acid (DNA) was isolated using the QIAamp DNA Mini and Blood Mini Kit (Qiagen) according to the manufacturer's instructions. Briefly, bacterial cells were harvested from culture plates, resuspended in 180 μL lysis buffer ATL, and incubated with 20 μL proteinase K at 56 °C for 1 h. Following lysis, 200 μL buffer AL was added, and samples were incubated at 70 °C for 10 min, after which 200 μL 96–100% ethanol was added. The lysate was applied to QIAamp spin columns and centrifuged at 8,000 rpm for 1 min, then washed sequentially with wash buffer AW1 and AW2 according to the kit protocol, and DNA was finally eluted in 40 μL elution buffer AE. DNA concentration and purity were subsequently determined using a Qubit fluorometer (Thermo Fisher Scientific) with the Qubit double-stranded DNA (dsDNA) assay kit, following the manufacturer's recommendations.

### 2.3 Whole-genome sequencing

Whole-genome sequencing libraries were. prepared using the MGIEasy Fast FS Library Prep Set V2.0 (MGI Tech Co., Ltd., Shenzhen, China) according to the manufacturer's instructions. Sequencing was performed on the DNBSEQ-G99RS platform (MGI Tech Co., Ltd., Shenzhen, China). High-quality genomic DNA was fragmented to the desired insert size and subjected to size selection to obtain a uniform library. Fragmented DNA underwent end repair and 3′-A tailing, after which sequencing adapters compatible with the DNBSEQ platform were ligated. Adapter-ligated products were cleaned by a second size selection step to remove undesired fragments and adapter dimers, followed by PCR amplification to enrich properly ligated molecules. Final libraries were evaluated for concentration and fragment size before sequencing on the DNBSEQ short-read platform using standard run conditions.

### 2.4 Genome assembly and annotation

Raw paired-end reads generated on the DNBSEQ platform were assessed with FastQC to evaluate base-quality profiles, GC content, and adapter contamination. Reads were trimmed and filtered with fastp using a minimum Phred quality score of 20, sliding-window mean quality of Q20, a minimum read length of 50 bp, and automatic paired-end adapter detection; quality metrics were recorded in JSON and HTML formats. Post-trimming quality was confirmed with a second FastQC run. High-quality reads were assembled *de novo* with SPAdes using the -careful flag to reduce mismatches and short indels. Assembly statistics including contig number, N50, genome size, and GC content were calculated with QUAST (Chen et al., 2018; Bankevich et al., 2012). Genomes were annotated with Prokka using kingdom Bacteria, genus *Streptomyces*, and Prodigal for gene prediction, generating standard output files (GFF, GBK, FAA, FFN) (Seemann, 2014).

### 2.5 Multi-tool cross-validation of biosynthetic gene clusters (BSGs)

To minimize method-specific false positives and to objectively rank candidate clusters, BGCs were predicted independently with three tools that implement fundamentally different detection paradigms, and their outputs were reconciled through a consensus scheme. antiSMASH (v8.0.4) (Blin et al., 2025) served as the primary, rule-based predictor, in which curated detection rules are applied to profile hidden Markov model (pHMM) hits against libraries of signature biosynthetic domains. GECCO (v0.10.2) (Carroll et al., 2021) provided an orthogonal, probabilistic prediction by modeling the Pfam protein-domain composition of each locus with a conditional random field, whereas DeepBGC (v0.1.28) (Hannigan et al., 2019) contributed a deep-learning prediction based on a bidirectional long short-term memory (BiLSTM) neural network operating on Pfam2vec domain embeddings. Because these approaches rely on independent sources of evidence expert-encoded rules, a domain-based statistical model, and a learned sequence representation their concordance provides a stringent test of the biological plausibility of a predicted cluster.

Each anti SMASH region was treated as the reference prediction and cross-checked for spatial overlap against the GECCO and Deep BGC predictions and was then assigned to one of three confidence tiers reflecting the degree of inter-tool agreement. Regions recovered by both GECCO and Deep BGC were designated HIGH confidence, those recovered by only one of the two tools were designated MEDIUM confidence, and anti SMASH-only regions supported by neither tool were designated LOW confidence. This tiering provided a transparent measure of prediction robustness and was used to prioritize candidates for downstream analysis, with HIGH-confidence regions considered the most reliable.

Annotated genome assemblies of the selected *Streptomyces* strains were imported into Geneious Prime^®^ 2026.0.2 (Ltd B., 2026) (Biomatters Ltd., Auckland, New Zealand) for genome visualization and comparative genomic analysis. Circular genome maps were generated to illustrate the distribution of BGCs and associated regulatory genes.

BGCs were initially identified using anti SMASH and subsequently mapped onto the corresponding genome assemblies in Geneious Prime (Kearse et al., 2012). Putative metal-responsive regulatory genes located within or adjacent to prioritized BGCs were identified based on functional annotations, conserved domain predictions, and sequence homology to previously characterized metal-responsive regulators, including members of the *Fur, MuR*, and *Zur*.

Genomic coordinates and spatial relationships between BGCs and putative metal-responsive regulators were manually inspected using the sequence viewer in Geneious Prime. Candidate BGCs were prioritized based on cryptic BGCs identified from known clusters deposited in the MIBiG database and their potential to encode previously uncharacterized specialized metabolites. Circular genome maps were subsequently generated to visualize the genome-wide distribution of prioritized untapped BGCs and their associated metal-responsive regulatory features.

### 2.6 *In silico* resistance screening and taxonomic classification

Antimicrobial resistance genes, virulence factors, and plasmid replicons were screened with ABRicate against the CARD, VFDB, and Plasmid Finder databases, and per-database summary reports were generated. For taxonomic validation, 16S rRNA gene sequences extracted from the Prokka annotations were queried by BLASTn against the NCBI nucleotide database, aligned with MAFFT (–auto –maxiterate 1,000), and used for maximum-likelihood phylogenetic tree reconstruction with FastTree under the GTR + GAMMA substitution model with 1,000 bootstrap replicates (Seemann, 2014; O'Leary et al., 2024; Katoh and Standley, 2013; Price et al., 2010; Alcock et al., 2023; Seemann, 2020; Carattoli et al., 2014). *Kitasatospora setae* was included as an outgroup. Whole-genome average nucleotide identity (ANI) was calculated using fastANI against a panel of reference genomes, with a 95% ANI threshold applied for species-level delineation (Jain et al., 2018). Outputs from all upstream analytical steps were collated with MultiQC to produce an integrated quality-control report (Ewels et al., 2016) (Supplementary Table 1).

### 2.7 Comparative genomic profiling and subtractive genome mining

A subtractive mining strategy was applied to identify BGCs present in *Streptomyces spp*. but absent from their corresponding reference genomes. BGC type matching between *Streptomyces spp*. and reference regions was determined using a Jaccard similarity coefficient, with a threshold of ≥0.5 defining shared clusters. Regions unique to the *Streptomyces spp* were retained as lineage-specific candidates. All antiSMASH-predicted regions were additionally scored against the MIBiG database of characterized clusters via Cluster Blast; regions with < 50% MIBiG similarity were classified as novel candidates. Cryptic BGCs were further categorized based on structural features, including uncommon biosynthetic classes (e.g., cyanobactin, thioamitides, beta-lactone, and hglE-KS), hybrid multi-class architecture, and large size (>80 kb) without conserved core biosynthetic domains (Supplementary Table 2).

Functional annotation, including KEGG pathway assignments, transporter classification (TCDB), drug target identification (TTD), and specialty gene detection (antibiotic resistance via PATRIC), was performed through the PATRIC/BV-BRC annotation server (Kanehisa, 2002). Genome visualization and manual inspection of BGC regions were conducted in Geneious Prime (Ltd B., 2026) (Supplementary Table 1).

## 3 Results

### 3.1 Phenotypic, taxonomic, and phylogenetic characterization of *Streptomyces spp*.

**Figure 1:**
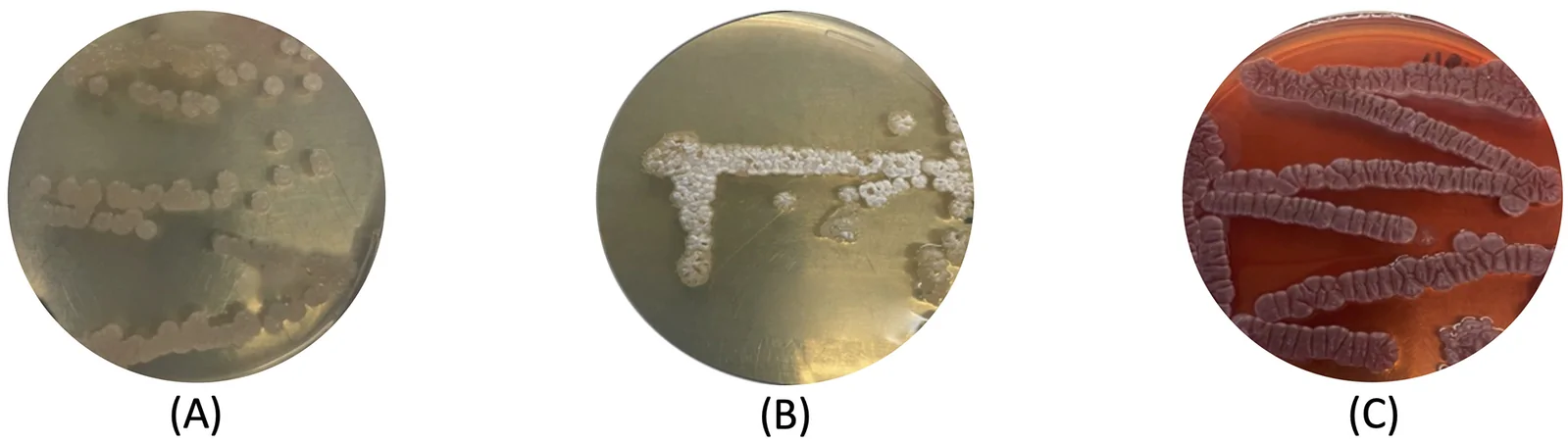
Colony morphology of the newly isolated Streptomyces spp., **(A)** *Streptomyces thinghirensis*, **(B)** *Streptomyces novocaesareae* **(C)** *Streptomyces griseorubens* grown on Mueller–Hinton agar (MHA) at 36°C for 7 days under aerobic conditions. The isolates exhibited distinct colony characteristics, including differences in pigmentation, surface texture, and aerial mycelium development. Colonies ranged from reddish rough-surfaced morphotypes to creamy smooth-surfaced and white dry rough-surfaced morphotypes, demonstrating phenotypic diversity among the isolates.

Purity confirmed by consistent colony morphology observed across successive subcultures. Following purification, the isolates (*Streptomyces spp*.) exhibited noticeable differences in colony morphology (Figure 1). Variations observed in pigmentation, texture, and surface appearance. Some isolates produced reddish colonies with a rough surface, whereas others formed creamy-colored colonies with a smooth surface. Additional isolates displayed white colonies with a rough and dry appearance. These phenotypic differences indicate morphological diversity at the colony level. However, colony morphology in *Streptomyces spp*. is strongly influenced by culture conditions and is generally insufficient for reliable species-level identification. Therefore, genome-based analyses employed to accurately determine taxonomic relationships and assess genetic differences among the *Streptomyces spp*.

The whole-genome assembly of newly isolated *Streptomyces spp*. demonstrated significant variations in assembly continuity and genome structure, yet all three preserved genomic characteristics typical of the genus displayed elevated GC contents between 71.2% and 72.4%, aligning with the typically GC-rich genomes documented for the genus *Streptomyces*, hence reinforcing their taxonomic classification within this group. *S. thinghirensis* demonstrated a genome size of 9.18 Mb with a fragmented genome assembly comprising 1,414 contigs (N50 = 338,596 bp; L50 = 10). *S. novocaesareae* exhibited the largest genome (10.82 Mb) and the highest assembly contiguity, comprising only 371 contigs and the highest N50 value (354,004 bp; L50 = 10) indicating either enhanced sequencing yield or actual genome growth compared to the other isolates. *S. griseorubens* displayed the smallest genome (7.75 Mb) and the highest GC content (72.43%), indicative of a more compact genomic structure; its N50 of 220,143 bp (L50 = 13) was inferior to those of isolates *S. thinghirensis* and *S. novocaesareae* (Supplementary Table 1).

Plasmid screening with PlasmidFinder revealed two replicons (ColRNAI_1 and IncR_1) in *S. thinghirensis*, while *S. novocaesareae*, and *S. griseorubens* exhibited no detectable plasmid sequences. No circular chromosomes were detected in any of the three assemblies. These parameters designate *S. novocaesareae*, as the most robust option for comprehensive genome mining and comparative biosynthetic study, whereas isolates *S. thinghirensis*, and *S. griseorubens* offer supplementary genetic diversity within the panel.

Core identification of the isolates was performed using a combined 16S ribosomal RNA (rRNA) gene sequence similarity and phylogenetic framework. Initial taxonomic assignment was carried out by BLASTn analysis of partial 16S rRNA gene sequences against the NCBI RefSeq database, with closest reference strains selected based on percentage identity, query coverage, and pairwise identity. All strains (*S. thinghirensis, S. novocaesareae*, and *S. griseorubens*) exhibited ≥99.0% sequence identity and ≥95% query coverage relative to validated *Streptomyces* reference strains, supporting robust genus-level classification (Supplementary Table 3).

To refine species-level relationships and mitigate potential misclassification due to the conserved nature of the 16S rRNA locus, phylogenetic analysis was performed using reference sequences corresponding to the top BLAST hits. Multiple sequence alignment was followed by construction of a distance-based phylogenetic tree, and node robustness was quantified using consensus support values. In all cases, each *spp*. grouped with its BLAST-identified reference species with high branch support (≥77–100%), demonstrating strong concordance between pairwise sequence similarity and evolutionary placement and confirming the assignment of each isolate to its nearest reference species (Supplementary Table 3).

**Figure 2:**
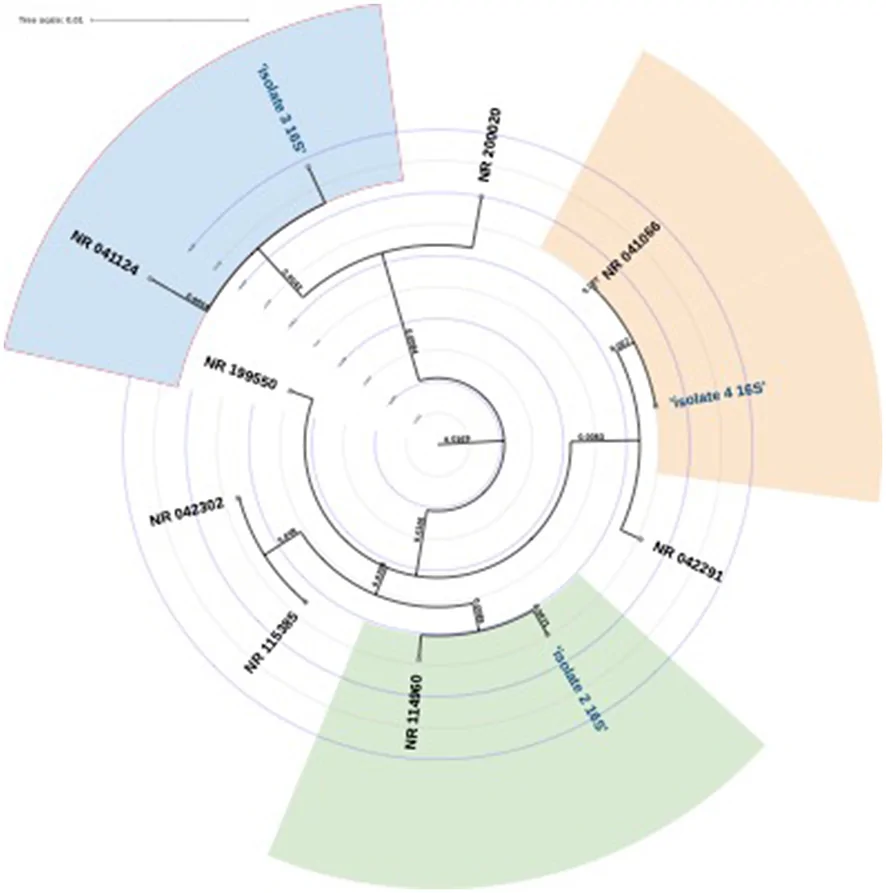
16S rRNA gene phylogeny of *Streptomyces* newly sequenced and reference strains. Rooted distance tree inferred from a nucleotide multiple-sequence alignment of strains *S. thinghirensis, S. novocaesareae*, and *S. griseorubens* 16S and their closest NCBI RefSeq reference sequences (consensus tree exported.newick). Branch lengths represent evolutionary distance (substitutions per site).

Phylogenetic analysis of partial 16S rRNA gene sequences showed strong evolutionary conservation among the isolates and reference *Streptomyces* strains and was concordant with BLASTn assignments. *S. thinghirensis* 16S clustered with *Streptomyces thinghirensis* (strain S10) with 98.2% consensus support, and *S. griseorubens* 16S grouped with *Streptomyces griseorubens* (strain NBRC 12780) with 100% support. Phylogenetic reconstruction of partial 16S rRNA gene sequences placed *S. novocaesareae* in a single clade with *Streptomyces novacaesareae* 16S (strain NBRC 13368), supporting its closest relationship to this reference lineage (Figure 2).

### 3.2 Multi-tool cross-validation of BGCc and consensus prioritization

**Figure 3:**
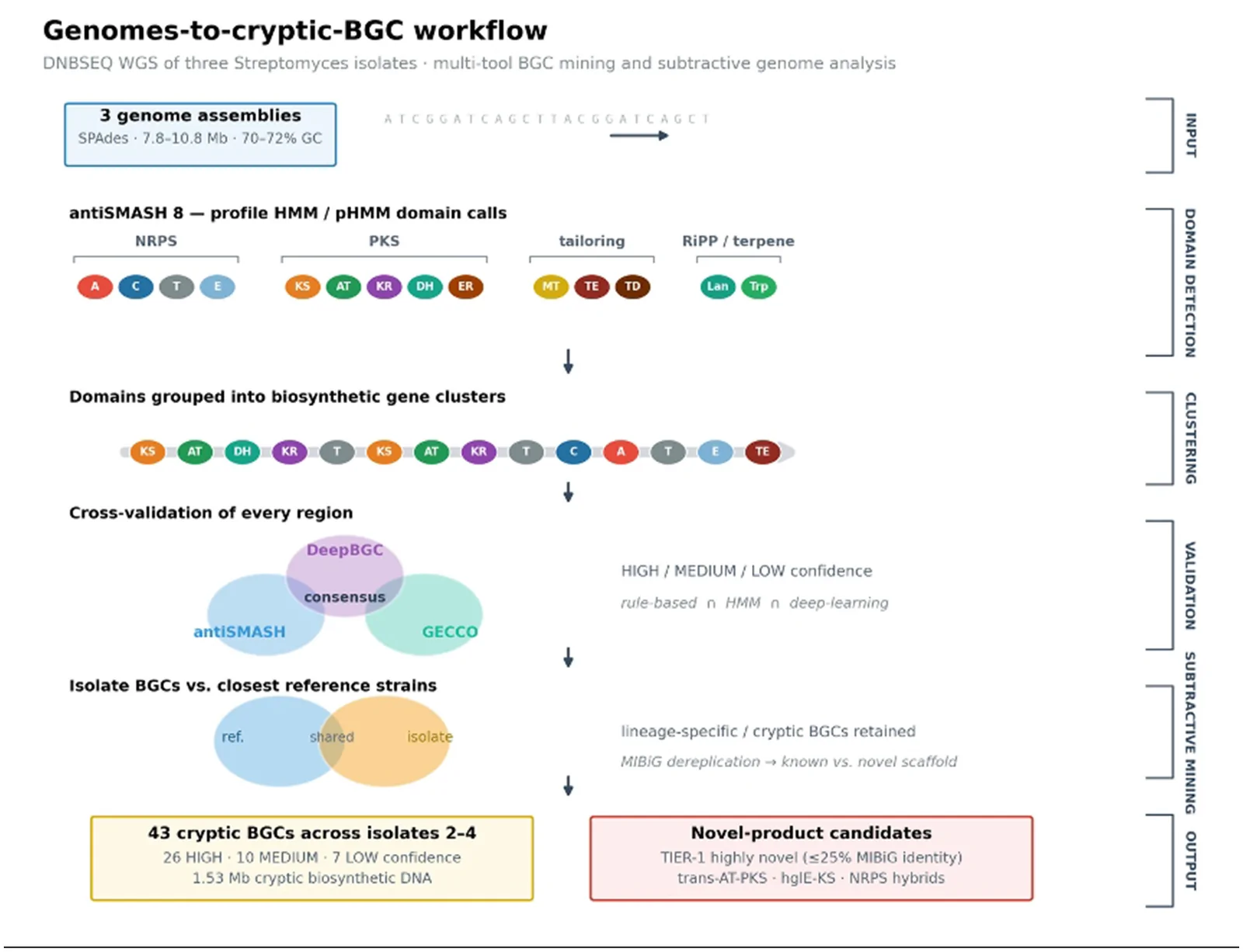
Workflow for the identification of cryptic BGCs from whole-genome sequencing data of three *Streptomyces* spp. using an integrated genome mining strategy. Predicted BGCs were identified with antiSMASH, DeepBGC, and GECCO, followed by subtractive genome analysis to prioritize cryptic clusters. Confidence levels were assigned based on agreement among prediction tools: **High confidence** indicates BGCs independently predicted by both GECCO and DeepBGC; **Medium confidence** indicates BGCs predicted by either GECCO or DeepBGC; and **Low confidence** indicates BGCs identified exclusively by antiSMASH. Among the 43 prioritized cryptic BGCs, 26 were classified as high confidence, 10 as medium confidence, and 7 as low confidence.

An integrated genome mining workflow combining antiSMASH, DeepBGC, and GECCO was employed to prioritize cryptic biosynthetic gene clusters (BGCs) from the three *Streptomyces* isolate genomes (Figure 3). Candidate BGCs were subsequently ranked according to the level of agreement among the prediction tools. BGCs independently identified by both DeepBGC and GECCO were classified as high confidence, those detected by either DeepBGC or GECCO were classified as medium confidence, whereas BGCs identified exclusively by antiSMASH were assigned low confidence.

Applying this consensus framework across the three *Streptomyces* genomes yielded 43 cryptic or low-similarity BGCs, of which 26 were classified as HIGH, 10 as MEDIUM, and 7 as LOW confidence. The predominance of high-confidence predictions indicates substantial agreement between complementary machine learning-based approaches and supports the robustness of the prioritized cryptic BGC repertoire. These high-confidence clusters were therefore considered the most promising candidates for downstream functional characterization and secondary metabolite discovery.

**Figure 4:**
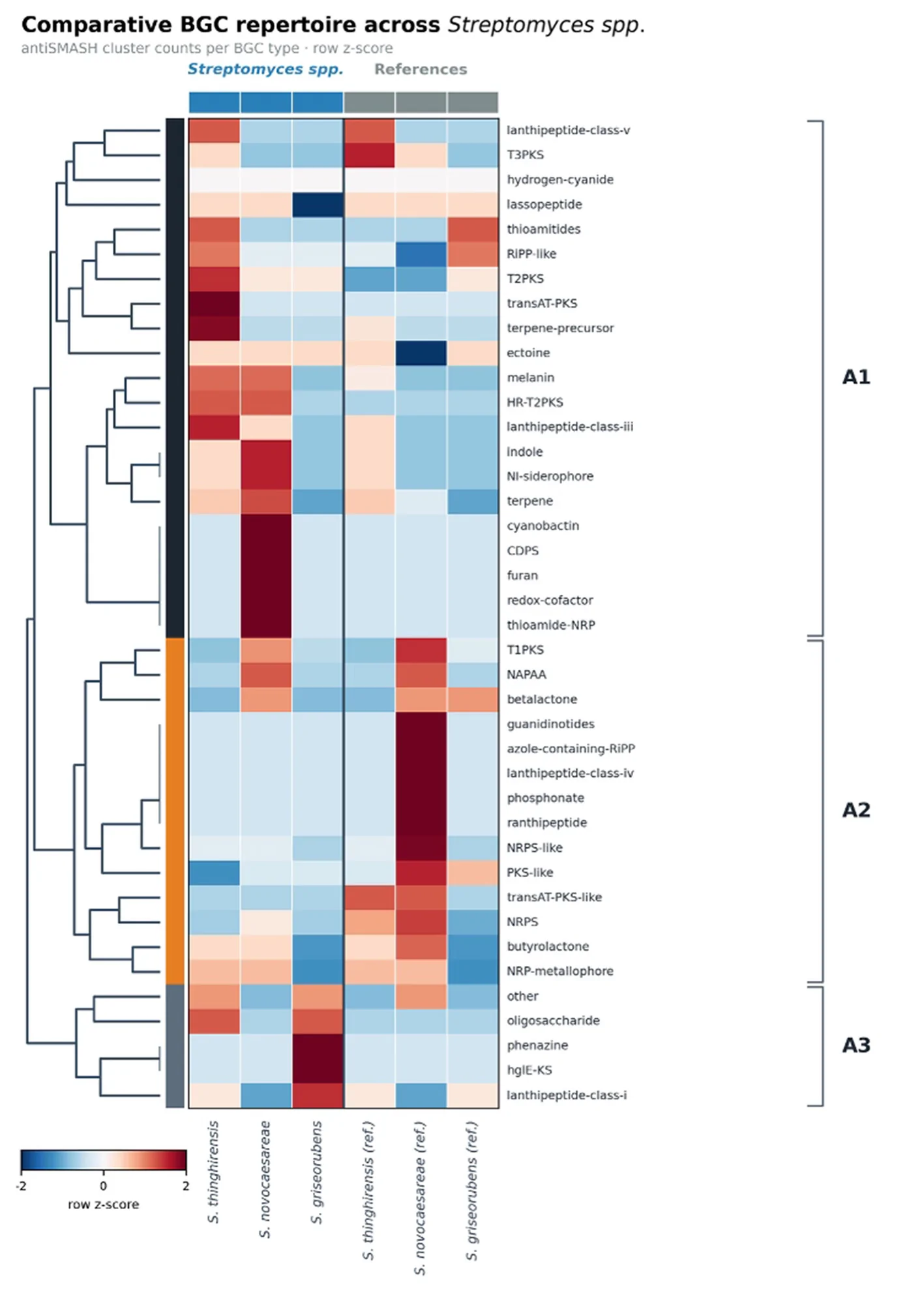
Comparative heatmap of BGCs classes across three investigated *Streptomyces spp*. and three reference genomes. Rows represent BGCs classes and columns represent genomes. Hierarchical clustering of row-wise z-score normalized BGC abundances grouped BGC classes into three data-driven clusters (A1–A3). The heatmap colors represent row-wise z-scores, where red indicates relatively higher abundance and blue indicates relatively lower abundance of a given BGC class compared with its mean abundance across all genomes.

Hierarchical clustering of row-wise normalized BGC abundances partitioned the BGC classes into three computationally derived clusters (A1–A3) based on similarities in their abundance profiles across the three investigated *Streptomyces spp*. and three reference genomes (Figure 4). These clusters represent statistical groupings of BGC classes with similar distribution patterns rather than predefined biological categories.

Cluster A1 comprised the largest and most heterogeneous group, containing widely distributed BGC classes shared among *Streptomyces spp*. and reference genomes, including terpene, melanin, NI-siderophore, RiPP-like, lanthipeptides, hydrogen cyanide, and type II and III polyketide synthases (T2PKS and T3PKS). This cluster also included several BGC classes that were preferentially enriched in *Streptomyces novocaesareae*, such as NAPAA, CDPS, furan, cyanobactin, redox-cofactor, and thioamide-NRP clusters.

Cluster A2 consisted primarily of BGC classes enriched in the reference genomes, particularly the *S. novocaesareae* reference strain, including guanidinotides, azole-containing RiPPs, class IV lanthipeptides, phosphonates, ranthipeptides, NRPS, PKS-like, trans-AT PKS-like, butyrolactones, and NRP-metallophore clusters.

Cluster A3 represented a small subset of BGC classes exhibiting sparse or lineage-specific distribution patterns, including oligosaccharide, phenazine, hglE-KS, and class I lanthipeptide clusters.

### 3.3 Comparative analysis of metal-responsive regulators in novel *Streptomyces spp*. genomes

Genome mining identified multiple putative metal-responsive regulators across the analyzed *Streptomyces spp*. genomes, including the ferric uptake regulator (*Fur*), zinc uptake regulator (*Zur*), and nickel uptake regulator (*NuR*). Genome mining analysis further revealed predicted *Fur, Zur*, and *NuR*-associated regulatory elements in proximity to diverse biosynthetic gene clusters, including NRPS, T1PKS, T2PKS, T3PKS, terpene, RiPP-like, and hydrogen-cyanide clusters. Comparative analysis indicated that these regulatory systems were distributed across all investigated isolates and were associated with biosynthetic regions encoding diverse specialized metabolite classes.

Comparative genome mining across *S. thinghirensis, S. novocaesareae*, and *S. griseorubens* identified a total of 43 cryptic BGCs, highlighting a substantial reservoir of unexplored biosynthetic potential. (39.4% of all 109 predicted regions), encompassing 1,525.7 kb of biosynthetic DNA and spanning 17 distinct cluster classes.

To enhance the discovery potential of bioactive natural product analogs from the newly sequenced strains (*S. thinghirensis, S. novocaesareae*, and *S. griseorubens)* the predicted BGCs were systematically categorized according to their degree of homology to previously characterized secondary metabolites via antiSMASH. In line with our focus on comparative genome mining of *Streptomyces spp*., each displaying notable bioactivities and harboring cryptic biosynthetic potential for novel drug analogs.

**Figure 5:**
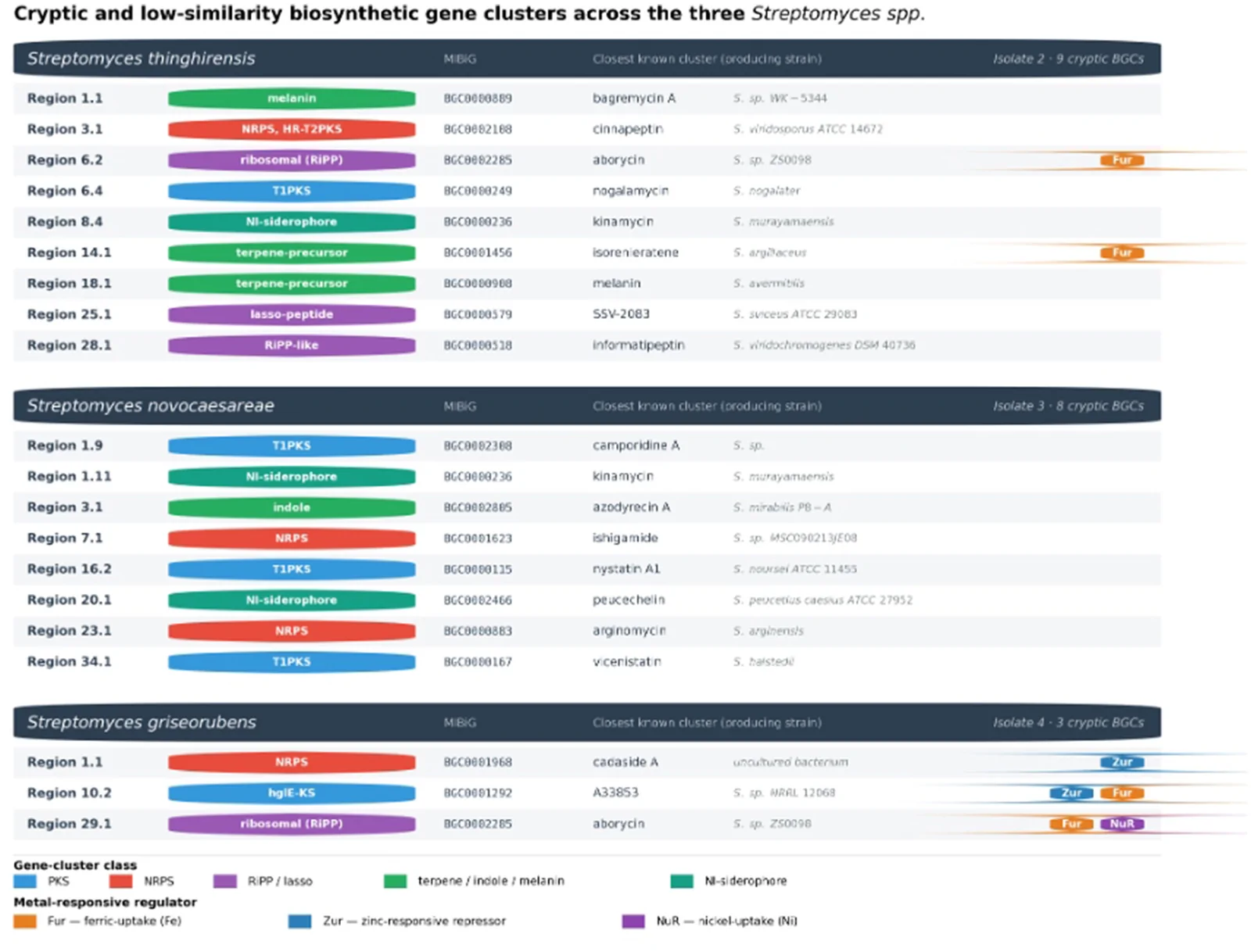
Integrated comparative analysis of BGCs, metal-responsive regulatory networks, and predicted secondary metabolite scaffolds in selected *Streptomyces* genomes.

Comparative analysis identified 9, 8, and 3 low-similarity and cryptic BGCs in *S. thinghirensis, S. novocaesareae*, and *S. griseorubens*, respectively (Figure 5).

Several of these clusters exhibited low similarity to characterized BGCs deposited in the MIBiG database, indicating limited correspondence to previously reported biosynthetic pathways. Putative metal-responsive regulators, including *Fur, Zur*, and *NuR*, were detected within or adjacent to two cryptic BGC in *S. thinghirensis*, and all three cryptic BGCs has been found linked to *Fur, Zur*, and *NuR* in corrospond to *S. griseorubens*, whereas no such associations were observed among the cryptic BGCs of *S. novocaesareae*.

**Figure 6:**
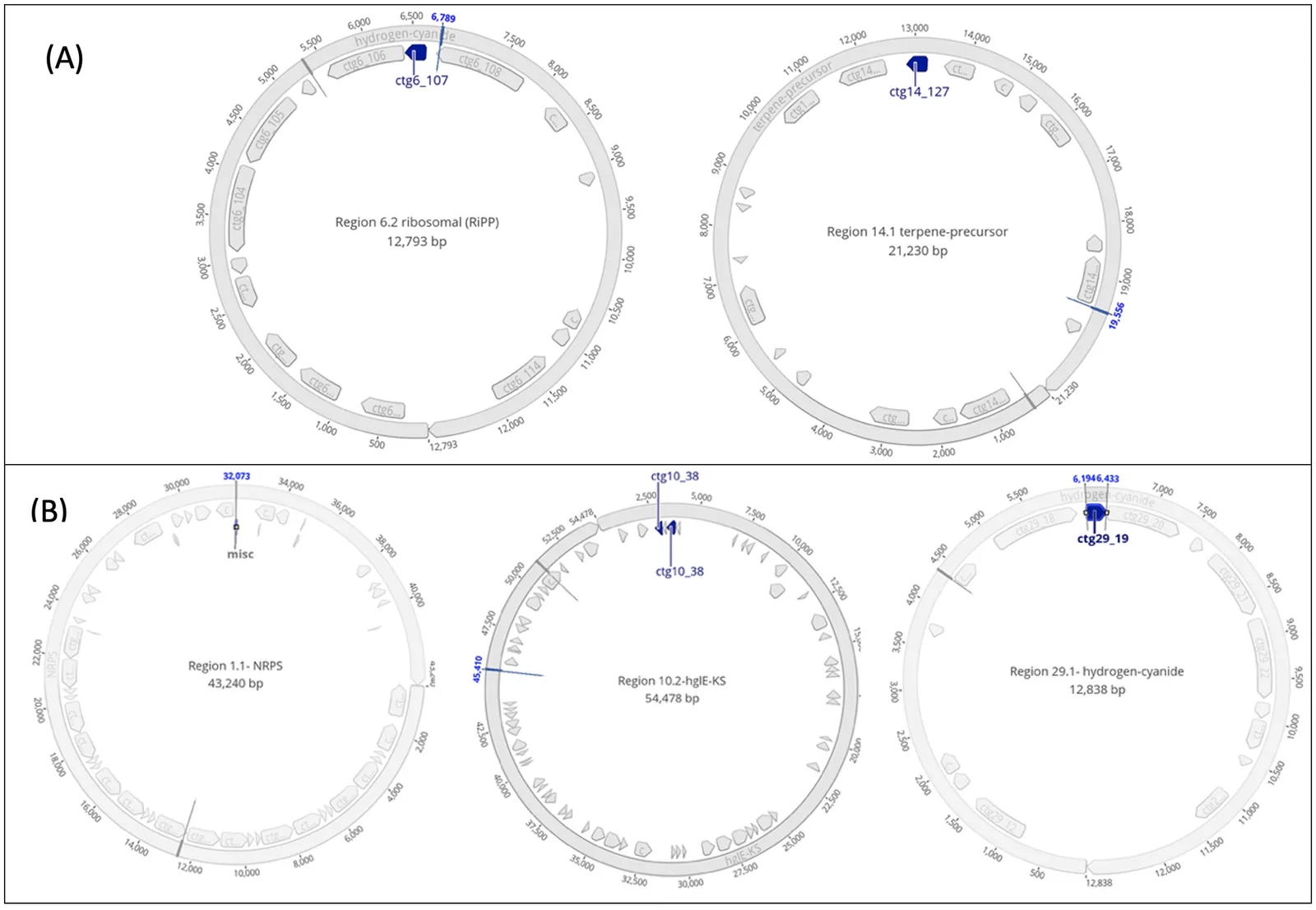
Genome-wide distribution of untapped BGCs and putative metal-responsive regulators in two *Streptomyces* genomes. **(A)** *S. thinghirensis* containing two prioritized untapped BGCs. **(B)** *S. griseorubens* containing three prioritized untapped BGCs. Highlighted regions indicate candidate clusters with potential for novel specialized metabolite biosynthesis.

Genome-wide visualization of the selected *Streptomyces spp*. genomes revealed the spatial distribution of prioritized untapped BGCs and their associated putative metal-responsive regulatory elements (Figure 6). Five candidate BGCs were prioritized based on their low similarity to characterized clusters in the MIBiG database and their potential to encode previously unreported specialized metabolites.

In *S. thinghirensis*, two prioritized BGCs were identified and were found to be associated with putative metal-responsive regulatory genes located within the cluster boundaries (Figure 6A marked with blue arrows). Similarly, three prioritized BGCs were identified in *S. griseorubens*, each exhibiting genomic proximity to predicted metal-responsive regulators (Figure 6B marked with blue arrows).

The observed co-localization of these regulatory elements with cryptic or low-similarity BGCs suggests a potential role for metal-dependent regulation in specialized metabolite biosynthesis. The presence of regulator-associated BGCs in both genomes further supports the hypothesis that metal-responsive signaling may contribute to the activation of otherwise silent or poorly expressed biosynthetic pathways. These findings highlight the potential of metal-based elicitation strategies for unlocking novel specialized metabolites encoded by these untapped genomic regions.

**Figure 7:**
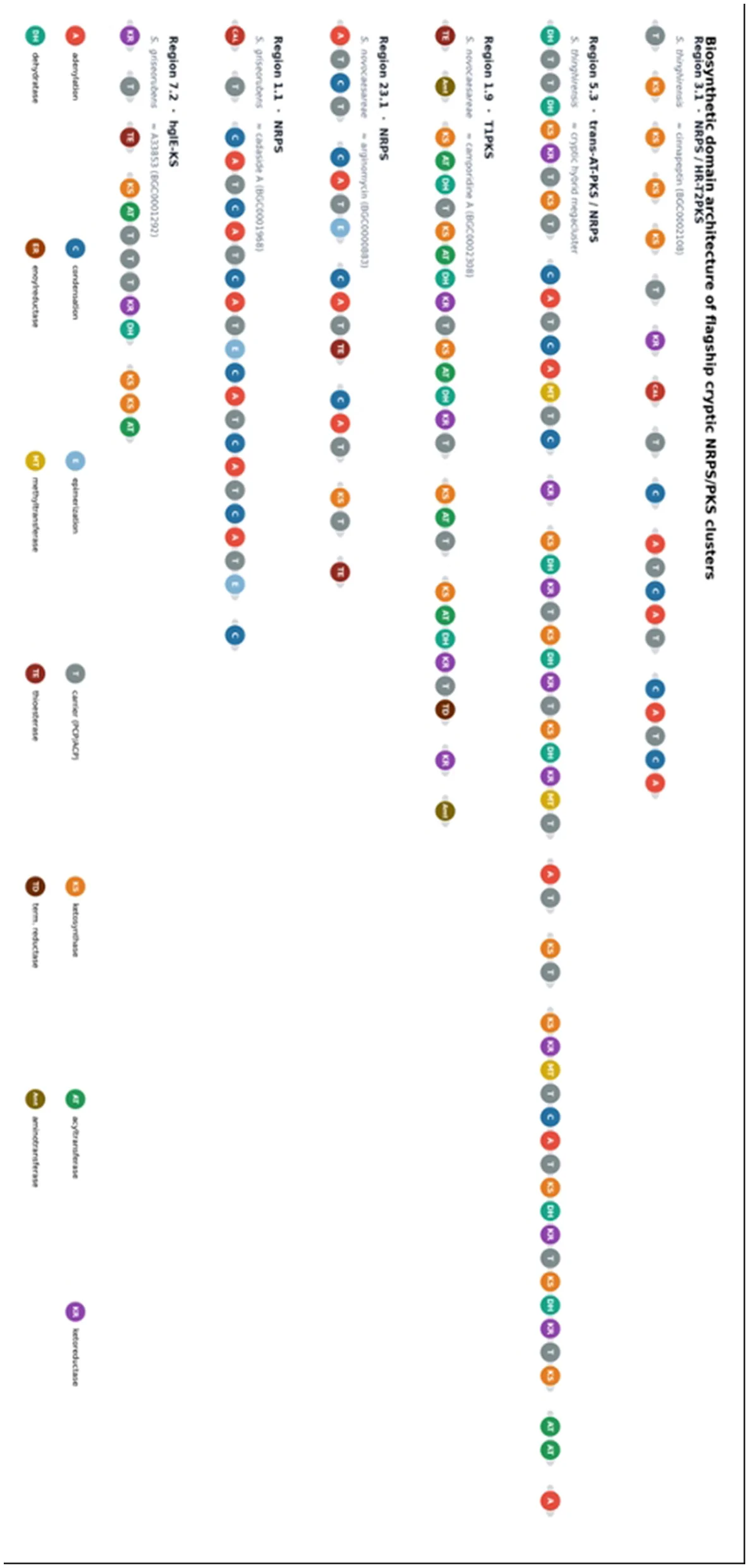
PRISM-based prediction of representative flagship cryptic BGCs identified in three newly *Streptomyces spp*. Representative cryptic BGCs prioritized through the consensus genome-mining workflow were further analyzed using PRISM to predict their biosynthetic domain architecture and associated metabolite scaffolds. The selected clusters comprise NRPS, trans-AT PKS, hybrid trans-AT PKS/NRPS, T1PKS, and hglE-KS pathways identified in *S. thinghirensis, S. novocaesareae*, and *S. griseorubens*. The closest homologous metabolites predicted by PRISM based on sequence similarity are indicated for each representative cluster.

PRISM analysis reconstructed the biosynthetic domain architectures of representative high-confidence cryptic BGCs, providing structural support for the predicted metabolite scaffolds (Figure 7). The selected clusters exhibited conserved catalytic domains characteristic of NRPS, PKS, and hybrid PKS–NRPS assembly lines.

In *S. thinghirensis*, the cinnapeptin-like NRPS/HR-T2PKS cluster contained KS, T, KR, and CAL domains, whereas the hybrid trans-AT PKS/NRPS cluster comprised DH, KS, KR, T, C, A, and MT domains, consistent with a modular peptide-polyketide assembly. The T1PKS cluster of *S. novocaesareae* displayed the canonical PKS architecture containing KS, AT, DH, KR, T, TE, and AmT domains, supporting its predicted camporidine A-like scaffold. In *S. griseorubens*, the cadaside A-like NRPS cluster consisted of the characteristic C, A, T, E, and TE domains, while the hglE-KS cluster contained KS, AT, KR, DH, T, and TE domains, consistent with a polyketide biosynthetic pathway.

## 4 Discussion

This study demonstrates the value of integrating complementary genome-mining platforms to systematically uncover the hidden biosynthetic potential of newly identified *Streptomyces spp*. Comparative analyses using antiSMASH, DeepBGC, GECCO, and PRISM revealed extensive biosynthetic diversity, numerous cryptic BGCs, and multiple putatively novel secondary metabolite scaffolds that would have been overlooked using a single prediction platform. The integration of multiple computational approaches further enabled consensus prioritization of cryptic BGCs, providing a robust framework for guiding downstream functional characterization and natural product discovery. The three newly *Streptomyces spp*. genomes exhibited assembly statistics consistent with the large, GC-rich genomes typical of the genus. Although isolates possessed a genome size comparable to previously reported *Streptomyces* species, its assembly remained highly fragmented. This likely reflects the abundance of repetitive genomic regions and complex secondary metabolite loci characteristic of *Streptomyces*, which frequently challenge short-read genome assembly. Similar assembly fragmentation has been reported in numerous *Streptomyces* genomes generated exclusively from Illumina sequencing, whereas hybrid or long-read sequencing approaches generally provide more contiguous assemblies and improved recovery of complete biosynthetic gene clusters.

This investigation integrated whole-genome sequencing of three newly bioactive *Streptomyces spp*. using the DNBSEQ short-read platform with comprehensive quality control, genome annotation, phylogenetic analysis, and systematic BGC discovery through comparative analysis using antiSMASH, GECCO, and DeepBGC. The identified BGCs were further contextualized through similarity assessment against the MIBiG database. The consensus-based genome-mining strategy improved confidence in the prioritization of cryptic biosynthetic gene clusters by integrating predictions from antiSMASH, DeepBGC, and GECCO. The predominance of high-confidence clusters (26 of 43) indicates substantial agreement among complementary prediction algorithms, supporting the robustness of the identified candidate BGCs. In contrast, medium- and low-confidence clusters may represent highly divergent biosynthetic pathways or tool-specific predictions that require further validation. These findings highlight the advantage of combining multiple genome-mining platforms to improve cryptic BGC discovery and provide a reliable foundation for subsequent metabolomic characterization and natural product discovery.

Furthermore, comparative genome analysis demonstrated that the investigated newly identified *Streptomyces spp*. possess metabolically rich and biosynthetically diverse genomes, containing a broad repertoire of BGCs associated with secondary metabolite production. The identified genomic features suggest a strong capacity for the biosynthesis of structurally diverse natural products with potential pharmaceutical relevance. Notably, *Streptomyces novacaesareae* has not previously been extensively explored in the context of genome-guided natural product discovery, highlighting the novelty of this study and the potential of this species as a promising source of previously uncharacterized bioactive compounds despite the lack of metal-responsive regulators.

Comparative analysis of BGC repertoires provides valuable insights into the evolutionary diversification of secondary metabolism in *Streptomyces spp*. Although closely related species often share a conserved core set of BGCs, lineage-specific gain, loss, and diversification of biosynthetic pathways contribute to strain-dependent metabolic capabilities and ecological adaptation. The hierarchical clustering observed in this study reflects these evolutionary patterns, distinguishing conserved BGC repertoires from strain-specific biosynthetic features. Notably, the enrichment of unique BGC classes in *Streptomyces novocaesareae* suggests an expanded biosynthetic capacity that may contribute to the production of previously uncharacterized specialized metabolites. These findings are consistent with previous studies demonstrating that comparative genome mining is an effective strategy for identifying lineage-specific biosynthetic diversity and prioritizing candidate clusters for natural product discovery (Hoskisson and Seipke, 2020; Raja and Subathra Devi, 2025).

Several cryptic BGCs exhibited low similarity to reference clusters identified by antiSMASH while receiving moderate-to-high confidence support from the consensus genome-mining strategy (Figure 5). These regions represent biosynthetic loci with limited similarity to previously characterized pathways but consistent prediction across multiple genome-mining platforms, suggesting the presence of potentially novel biosynthetic architectures or previously uncharacterized members of established biosynthetic families.

Comparative analysis further revealed distinct associations between cryptic BGCs and putative metal-responsive regulatory systems among the three *Streptomyces spp*. In *S. thinghirensis, Fur* was associated with cryptic RiPP and terpene BGCs showing similarity to the aborycin and isorenieratene biosynthetic pathways, respectively (Becerril et al., 2018; Shao et al., 2019). In *S. novocaesareae*, no putative metal-responsive regulators were identified in association with the prioritized cryptic BGCs, despite this isolate harboring eight cryptic clusters. In contrast, *S. griseorubens* exhibited the most diverse regulatory profile, with *Fur, Zur*, and *Nur* associated with multiple cryptic BGC classes, including NRPS, hglE-KS, and RiPP clusters showing similarity to the cadaside A, A33853, and aborycin biosynthetic pathways (Shao et al., 2019; Wu et al., 2019; Lv et al., 2015). Overall, considerable variation was observed in both the abundance of cryptic BGCs and their associated metal-responsive regulatory elements, highlighting substantial inter-isolate differences in their predicted biosynthetic and regulatory repertoires.

Collectively, these findings position the studied three new *Streptomyces spp*. as promising bio-factories for the identification of structurally modified or entirely novel compounds that are genetically and biosynthetically distinct from their closest reference counterparts.

Interestingly, putative metal-responsive regulators were associated with both characterized and cryptic BGCs. While several *Fur, Zu-*, and *Nur*-associated regions corresponded to BGCs with established biosynthetic signatures, others were linked to cryptic clusters exhibiting low similarity to known pathways. The association of metal-responsive regulatory elements with these cryptic BGCs suggests that metal-dependent regulation may influence the expression of otherwise silent biosynthetic pathways, highlighting promising targets for future activation strategies and natural product discovery (Romero-Rodríguez et al., 2015).

These observations are consistent with previous studies demonstrating that metal-responsive regulators play important roles in coordinating secondary metabolism and biosynthetic gene cluster expression in *Streptomyces* (Gu and Codd, 2012). For example, the strong copper-bleomycin interaction in *S. verticillus* highlights the potential of copper-based strategies for discovering potent anticancer metabolites, including improved analogs such as NC0604 (Gu and Codd, 2012). Transition-metal-ion stress in *S. coelicolor* M145 markedly altered the secondary metabolite profile, inducing prodigiosin biosynthesis and promoting the formation of novel derivatives (coeligiosins A and B) (Morgenstern et al., 2015). Similarly, stress-responsive regulatory networks in *S. coelicolor* were associated with enhanced antibiotic production, supporting the role of environmental stress in activating specialized metabolic pathways (Clara et al., 2015).

The agreement between PRISM-derived domain architectures and the metabolite scaffolds predicted from genome mining provides additional confidence in the annotation of prioritized cryptic BGCs. Integrating domain architecture reconstruction with complementary genome-mining approaches facilitates the prioritization of candidate biosynthetic pathways for downstream functional characterization and natural product discovery (Skinnider et al., 2015).

Particularly noteworthy was the association of putative metal-responsive regulators with cryptic BGCs. In *S. thinghirensis*, two of nine cryptic BGCs were associated with *Fur*, whereas all three prioritized cryptic BGCs in *S. griseorubens* were associated with *Fur, Zur*, and *Nur*. No comparable regulatory associations were detected in *S. novocaesareae*. Although these associations require experimental validation, they suggest that metal-responsive regulators may influence the expression of selected cryptic BGCs, providing promising targets for future activation and natural product discovery.

The occurrence of cryptic BGCs across our investigated *Streptomyces* genomes highlights their potential as reservoirs of previously unexplored biosynthetic diversity. Notably, several of these BGCs were associated with putative metal-responsive regulators, suggesting that metal-dependent regulatory networks may contribute to controlling specialized metabolite biosynthesis. Distinct CRISPR-based strategies tackle different limitations in actinomycete genome engineering; one system enables efficient gene/cluster deletion, precise HDR-based editing, and reversible silencing, achieving near 100% precision as demonstrated in the actinorhodin cluster of *S. coelicolor* (Tong et al., 2015), while Kim et al. (2025) developed Cas9-BD, a low-toxicity Cas9 variant that overcomes off-target cytotoxicity in high-GC *Streptomyces* genomes to enable multiplexed BGC editing and large-cluster capture. Such approaches could be applied to manipulate the putative metal-responsive regulators identified in the present study, thereby activating silent biosynthetic pathways or increasing metabolite yields. In parallel, synthetic biology strategies, including promoter engineering, heterologous expression (Wang et al., 2026), and chassis optimization, together with nanoparticle-mediated metabolic modulation, previously demonstrated to stimulate secondary metabolite biosynthesis, may provide complementary approaches for industrial-scale production of unexplored and novel natural products. Integrating genome mining approaches with these emerging genome-engineering technologies represents a promising strategy for accelerating the discovery, and enhancing the sustainable biosynthesis, of novel or higher-yielding bioactive compounds from bioactive *Streptomyces species*.

## Data Availability

All sequencing data generated in this study have been deposited in the National Center for Biotechnology Information (NCBI) under BioProject accession PRJNA1431580: https://www.ncbi.nlm.nih.gov/bioproject/PRJNA1431580.
