# Supplementary material for: Comparative genomics reveals hidden biosynthetic diversity in Streptomyces spp. and metal-dependent regulatory features associated with untapped specialized metabolites
Source: Front Microbiol. 2026 Aug 27;17:1928796. doi: 10.3389/fmicb.2026.1928796 (PMC13559783; doi:10.3389/fmicb.2026.1928796)
Supplement: Supplementary file 1 [file Table_1.DOCX]

Supplementary Material

# Supplementary Data

All sequencing data generated in this study have been deposited in the National Center for Biotechnology Information (NCBI) under BioProject accession PRJNA1431580. Raw sequencing reads are available in the Sequence Read Archive (SRA) under the associated experiment records linked to this BioProject. Genome assemblies and annotations, where applicable, are deposited in GenBank and are connected to the corresponding BioSample records. All data are publicly accessible via the BioProject page: <https://www.ncbi.nlm.nih.gov/bioproject/PRJNA1431580>

# 1 Supplementary Tables

Table S1: Integrated genomic, functional, and resistance features of *Streptomyces spp.**.

| **Category** | **Feature** | ***Streptomyces thinghirensis*** | ***Streptomyces novocaesareae*,** | ***Streptomyces griseorubens*** |
| --- | --- | --- | --- | --- |
| **Genome assembly** | Genome size (Mb) | 9.18 | 10.82 | 7.75 |
|  | GC content (%) | 71.18 | 70.73 | 72.43 |
|  | Contigs (n) | 1,414 | 371 | 348 |
|  | N50 (bp) | 338,596 | 354,004 | 220,143 |
| **Genome annotation** | Protein-coding genes (CDS) | 9,063 | 10,061 | 7,253 |
|  | tRNA genes | 76 | 81 | 72 |
|  | rRNA genes | 5 | 3 | 4 |
| **Protein annotation** | Hypothetical proteins | 2,774 | 3,428 | 2,099 |
|  | Proteins with functional assignment | 6,289 | 6,633 | 5,154 |
|  | KEGG-annotated proteins | 1,169 | 1,285 | 997 |
| **Specialty genes** | Antibiotic resistance (CARD) | 9 | 9 | 6 |
|  | Antibiotic resistance (PATRIC) | 60 | 54 | 47 |
|  | Transporters (TCDB) | 171 | 107 | 88 |
|  | Drug targets (TTD) | 6 | 3 | 3 |
| **AMR mechanisms** | Efflux systems | CmlV, Otr(C) | Otr(C) | CmlR, Otr(C) |
|  | Target modification | Erm family | Erm family | Erm family |
|  | Cell-wall modification | MprF, VanJ | MprF, VanJ | PgsA only |
|  | Additional features | Tet, FadG-like | AAC/APH enzymes | Streamlined profile |

***Assembly statistics from QUAST; gene annotation and functional categorization (CDS, hypothetical proteins, KEGG, TCDB, TTD, PATRIC specialty genes) from PATRIC/BV-BRC; antibiotic resistance gene counts (CARD row) from ABRicate using the CARD database.**

Table S2: Novel BGC candidates (<50% MIBiG similarity) across *Streptomyces spp.*

| **strains** | **Region ID** | **Contig** | **Position** | **Length (kb)** | **BGC type** | ***Confidence** | **MIBiG similarity (%)** |
| --- | --- | --- | --- | --- | --- | --- | --- |
| ***S.thinghirensis*** | c1_region1 | c1 | 271,148–281,523 | 10.4 | melanin | HIGH | 100 |
|  | c5_region3 | c5 | 222,479–327,849 | 105.4 | transAT-PKS, NRPS, NRPS-like | HIGH | 90 |
|  | c6_region1 | c6 | 109,143–119,725 | 10.6 | melanin | HIGH | 100 |
|  | c8_region2 | c8 | 137,937–149,314 | 11.4 | RiPP-like | LOW | 100 |
|  | c8_region3 | c8 | 282,548–293,598 | 11.1 | butyrolactone | HIGH | 66 |
|  | c5_region2 | c5 | 115,114–127,907 | 12.8 | hydrogen-cyanide | LOW | 100 |
|  | c10_region3 | c10 | 331,401–338,596 | 7.2 | butyrolactone | MEDIUM | 12 |
|  | c12_region3 | c12 | 233,411–306,160 | 72.7 | transAT-PKS, thioamitides | HIGH | 58 |
|  | c15_region2 | c15 | 135,644–156,772 | 21.1 | indole | HIGH | 100 |
|  | c16_region1 | c16 | 105,777–128,453 | 22.7 | lanthipeptide-class-iii, RiPP-like | HIGH | 80 |
|  | c17_region1 | c17 | 0–65,321 | 65.3 | T2PKS, oligosaccharide, other | HIGH | 80 |
|  | c25_region1 | c25 | 46,260–68,698 | 22.4 | lassopeptide | HIGH | 39 |
|  | c28_region1 | c28 | 7,582–17,798 | 10.2 | RiPP-like | LOW | 100 |
|  | c54_region1 | c54 | 0–8,618 | 8.6 | RiPP-like | MEDIUM | 27 |
| ***S. novocaesareae*,** | c4_region1 | c4 | 21,583–185,224 | 163.6 | NRPS, T1PKS, terpene-precursor, NRP-metallophore | HIGH | 100 |
|  | c6_region1 | c6 | 268,418–376,631 | 108.2 | T1PKS | HIGH | 35 |
|  | c19_region1 | c19 | 48,488–153,308 | 104.8 | NRPS, T3PKS | HIGH | 85 |
|  | c34_region1 | c34 | 0–89,028 | 89.0 | T1PKS | HIGH | 42 |
|  | c2_region1 | c2 | 363,454–418,353 | 54.9 | furan, PKS-like | HIGH | 82 |
|  | c7_region1 | c7 | 0–41,402 | 41.4 | thioamide-NRP, NRPS | HIGH | 51 |
|  | c13_region2 | c13 | 222,667–260,803 | 38.1 | terpene, melanin | HIGH | 100 |
|  | c1_region5 | c1 | 784,682–819,808 | 35.1 | NAPAA | HIGH | 100 |
|  | c22_region1 | c22 | 138,912–171,922 | 33.0 | HR-T2PKS, NRPS, NRPS-like | HIGH | 37 |
|  | c16_region1 | c16 | 15,727–43,114 | 27.4 | lanthipeptide-class-iii, RiPP-like | HIGH | 100 |
|  | c1_region8 | c1 | 1,179,111–1,204,560 | 25.4 | betalactone | MEDIUM | 95 |
|  | c1_region6 | c1 | 962,273–987,421 | 25.1 | terpene, butyrolactone | MEDIUM | 100 |
|  | c25_region1 | c25 | 0–23,629 | 23.6 | NAPAA | MEDIUM | 68 |
|  | c1_region3 | c1 | 514,026–536,572 | 22.5 | lassopeptide | HIGH | 100 |
|  | c26_region1 | c26 | 50,752–73,296 | 22.5 | cyanobactin | HIGH | 2 |
|  | c9_region1 | c9 | 125,789–147,926 | 22.1 | redox-cofactor | MEDIUM | 35 |
|  | c30_region1 | c30 | 87,408–108,659 | 21.3 | indole | MEDIUM | 76 |
|  | c3_region1 | c3 | 291,737–312,874 | 21.1 | indole | HIGH | 100 |
|  | c45_region1 | c45 | 15,145–28,742 | 13.6 | melanin, butyrolactone | HIGH | 25 |
|  | c1_region2 | c1 | 165,702–178,531 | 12.8 | hydrogen-cyanide | LOW | 92 |
|  | c32_region1 | c32 | 4,446–16,372 | 11.9 | RiPP-like | MEDIUM | 60 |
|  | c1_region7 | c1 | 1,074,169–1,085,567 | 11.4 | RiPP-like | LOW | 100 |
| ***S.griseorubens*** | c3_region2 | c3 | 70,961–163,400 | 92.4 | oligosaccharide, T2PKS, PKS-like | HIGH | 44 |
|  | c7_region2 | c7 | 47,768–102,246 | 54.5 | hglE-KS | HIGH | 48 |
|  | c31_region1 | c31 | 12,270–25,108 | 12.8 | hydrogen-cyanide | LOW | 100 |
|  | c48_region1 | c48 | 0–11,940 | 11.9 | phenazine | MEDIUM | 14 |
|  | c28_region1 | c28 | 83,534–94,914 | 11.4 | RiPP-like | HIGH | 100 |
|  | c1_region3 | c1 | 262,167–273,478 | 11.3 | RiPP-like | LOW | 88 |
|  | c2_region1 | c2 | 67,662–77,878 | 10.2 | RiPP-like | MEDIUM | 100 |

***Confidence: HIGH = supported by both GECCO and DeepBGC; MEDIUM = one tool; LOW = neither tool. MIBiG similarity = best antiSMASH ClusterBlast match (%).**

Table 4: Novel BGC candidates (<50% MIBiG similarity) across Streptomyces spp.

| **Strains** | **Region ID** | **BGC type** | **Length (kb)** | **MIBiG similarity (%)** | **Confidence** | **Closest known genome match** |
| --- | --- | --- | --- | --- | --- | --- |
| ***Streptomyces thinghirensis*** | c25_region1 | lassopeptide | 22.4 | 39 | HIGH | S. lincolnensis L4 |
| ***Streptomyces novocaesareae*,** | c26_region1 | cyanobactin | 22.5 | 2 | HIGH | S. sp. EAS-AB2608 |
|  | c1_region7 | terpene | 21.0 | 20 | LOW | S. cyaneochromogenes MK-45 |
|  | c45_region1 | melanin, butyrolactone | 13.6 | 25 | HIGH | S. sp. HUAS 31 |
|  | c5_region1 | terpene-precursor | 21.1 | 25 | MEDIUM | S. sp. FXJ1.172 |
|  | c6_region1 | T1PKS | 108.2 | 35 | HIGH | S. chartreusis NA02069 |
|  | c9_region1 | redox-cofactor | 22.1 | 35 | MEDIUM | S. chartreusis NA02069 |
|  | c22_region1 | HR-T2PKS, NRPS | 33.0 | 37 | HIGH | S. sp. HUAS 31 |
|  | c34_region1 | T1PKS | 89.0 | 42 | HIGH | S. chartreusis ATCC 14922 |
|  | c13_region1 | terpene | 21.0 | 43 | HIGH | S. ferrugineus CCTCC AA2014009 |
|  | c16_region2 | T1PKS | 23.2 | 46 | HIGH | S. sp. HUAS 31 |
|  | c48_region1 | T1PKS | 8.5 | 0 | LOW | No match |
|  | c50_region1 | NRPS | 3.7 | 0 | LOW | No match |
|  | c51_region1 | T1PKS | 3.7 | 0 | LOW | No match |
| ***Streptomyces griseorubens*** | c48_region1 | phenazine | 11.9 | 14 | MEDIUM | T. tengchongensis DSM 45615 |
|  | c3_region2 | T2PKS hybrid | 92.4 | 44 | HIGH | S. kanamyceticus ATCC 12853 |
|  | c7_region2 | hglE-KS | 54.5 | 48 | HIGH | S. fungicidicus F10 |

Table S3: Top five BLASTn hits (NCBI RefSeq rRNA) for 16S rRNA gene sequences of new isolates*.

| **Isolate** | **Rank** | **Accession (RefSeq)** | **Closest reference (description)** | **% Identical sites** | **Query coverage** | **% Pairwise identity** | **Hit length (bp)** |
| --- | --- | --- | --- | --- | --- | --- | --- |
| **(A)** | **1** | **NR_116901** | **Streptomyces thinghirensis strain S10, 16S rRNA (partial)** | **100.0%** | **95.93%** | **100.0%** | **1462** |
|  | 2 | NR_114960 | Streptomyces marokkonensis strain LMG 23016, 16S rRNA (partial) | 99.7% | 97.05% | 99.7% | 1479 |
|  | 3 | NR_115385 | Streptomyces aurantigriseus strain CSSP525, 16S rRNA (partial) | 99.3% | 98.10% | 99.3% | 1495 |
|  | 4 | NR_042302 | Streptomyces linemycini strain LMG 20091, 16S rRNA (partial) | 99.3% | 96.33% | 99.3% | 1468 |
|  | 5 | NR_199550 | Streptomyces okerensis strain DSM 116494, 16S rRNA (complete) | 99.2% | 100.0% | 99.2% | 1527 |
| **(B)** | **1** | **NR_041124** | **Streptomyces novacaesareae strain NBRC 13368, 16S rRNA (partial)** | **99.5%** | **96.91%** | **99.5%** | **1478** |
|  | 2 | NR_041216 | Streptomyces chartreusis strain NBRC 12753, 16S rRNA (partial) | 99.3% | 96.98% | 99.3% | 1477 |
|  | 3 | NR_200020 | Streptomyces machairae strain MS1.AVA.1, 16S rRNA (complete) | 99.3% | 100.0% | 99.3% | 1527 |
|  | 4 | NR_040857 | Streptomyces gallilaeus strain JCM 4757, 16S rRNA (partial) | 99.1% | 97.44% | 99.1% | 1484 |
|  | 5 | NR_115966 | Streptomyces aurantiacus strain NRRL B-3375, 16S rRNA (partial) | 99.1% | 98.56% | 99.1% | 1548 |
| **(C)** | **1** | **NR_041066** | **Streptomyces griseorubens strain NBRC 12780, 16S rRNA (partial)** | **100.0%** | **96.91%** | **100.0%** | **1476** |
|  | 2 | NR_178847 | Streptomyces euryhalinus strain MS 3/20, 16S rRNA (partial) | 99.7% | 98.56% | 99.7% | 1503 |
|  | 3 | NR_042290 | Streptomyces griseoincarnatus strain LMG 19316, 16S rRNA (partial) | 99.7% | 97.05% | 99.7% | 1480 |
|  | 4 | NR_112312 | Streptomyces griseoincarnatus strain NBRC 12871, 16S rRNA (partial) | 99.7% | 96.98% | 99.7% | 1479 |
|  | 5 | NR_042291 | Streptomyces griseoflavus strain LMG 19344, 16S rRNA (partial) | 99.7% | 97.05% | 99.7% | 1478 |

* BLASTn searches of the 16S rRNA gene sequences were performed in Geneious Prime against the NCBI RefSeq rRNA database, and the closest reference strains were selected based on percent identity, query coverage, and pairwise identity; the top five matches per isolate were retained for reporting.
